# QingGan LiDan capsules improved alcoholic liver injury by regulating liver lipid transport and oxidative stress in mice

**DOI:** 10.3389/fphar.2025.1575280

**Published:** 2025-03-26

**Authors:** Zhiwen Fu, Jiafeng Zhou, Hongye Pan, Song Yang, Zhenzhen Pan, Yujia Shen, Jianbiao Yao, Jiangning Hu

**Affiliations:** ^1^ School of Pharmaceutical Sciences, Zhejiang Chinese Medical University, Hangzhou, China; ^2^ Jiangxi Conba Traditional Chinese Medicine Co.,Ltd, Shangrao, China; ^3^ Zhejiang Conba Pharmaceutical Co.,Ltd, Hangzhou, China; ^4^ Zhejiang Provincial Key Laboratory of Traditional Chinese Medicine Pharmaceutical Technology, Hangzhou, China; ^5^ Zhejiang Key Laboratory of Major TCM Cultivation and TCM Innovation, Hangzhou, China

**Keywords:** QingGan LiDan capsule, alcoholic liver injury, liver function, lipid transport, oxidative stress

## Abstract

**Background:**

The QingGan LiDan capsule (QGLD) consists of five traditional Chinese herbs, which have been used for hepatobiliary diseases such as jaundice. However, the effects and mechanisms by which QGLD prevent alcoholic liver diseases (ALD) remain unknown.

**Aim of the study:**

Investigate the therapeutic potential of QingGan Lidan capsule (QGLD) in alleviating alcohol-induced liver injury.

**Materials and Methods:**

Acute alcoholic liver injury model and chronic and Binge ethanol Feeding Model (NIAAA) model were established. Mice were administered QGLD (360, 720, 1,440 mg/kg) or vehicle. Liver function indicators (ALT, AST), serum lipid (TC, TG), antioxidant markers (SOD, GSH, MDA), lipid metabolism/transport genes relative expression levels, liver and ileal villus morphology were analyzed. Network pharmacology analysis was also performed to identify potential targets and pathways of QGLD.

**Results:**

QGLD reduced serum ALT, AST, hepatic TC, TG, and lipid droplet accumulation in both models. It upregulated antioxidant enzymes (SOD, GSH) and downregulated MDA. QGLD regulated the mRNA levels of genes related to the NRF2/KEAP1 pathway and lipid transport. Network pharmacology identified 221 potential targets.

**Conclusion:**

QGLD mitigates alcohol-induced liver injury by reducing lipid accumulation, regulating lipid transport and enhancing antioxidant capacity. This supports its potential application in ALD management.

## Graphical Abstract

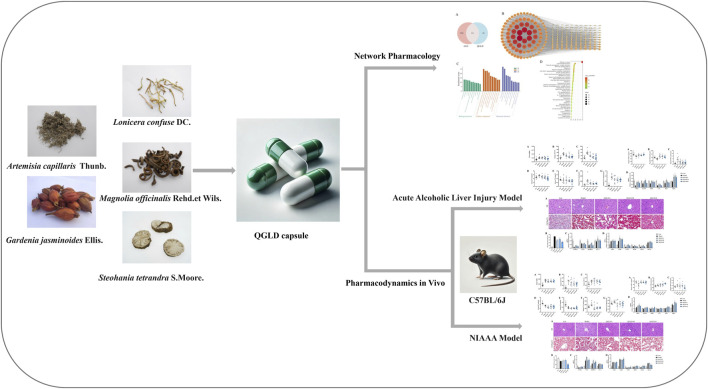

## 1 Introduction

Alcohol consumption is present in the majority of cultures worldwide, and alcohol abuse is a common problem globally. Excessive alcohol consumption can damage multiple organs, notably the brain, heart, gastrointestinal system, and liver (Evangelou et al., 2021). Alcoholic liver disease (ALD) stands as the foremost cause of liver-related ailments globally, characterized by a sequence of harmful transformations in the liver due to prolonged alcohol use, presenting as issues such as fatty liver and hepatitis (Yan et al., 2023). It encompasses a spectrum of progressive morphological changes, such as simple steatosis, hepatitis, steatohepatitis, and fibrosis. It can also lead to the progression to cirrhosis and an increased risk of hepatocellular carcinoma (Ventura-Cots et al., 2022). In the body, the ingested ethanol is primarily metabolized in the liver, where it is first transformed into acetaldehyde through alcohol dehydrogenase (ADH), and rapidly converted to acetate through aldehyde dehydrogenase (ALDH) (Hyun et al., 2021). The generation of ROS is closely related to the metabolism of alcohol to acetaldehyde and acetate, and acetaldehyde can induce the production of ROS (Salete-Granado et al., 2023). Oxidative stress caused by alcohol consumption disrupts the function of essential antioxidants such as glutathione peroxidase (GPX), superoxide dismutase (SOD) and catalase (CAT). Alcoholic fatty liver refers to the buildup of fat in liver cells without significant inflammation or liver fibrosis, which is seen in 90% of heavy drinkers (Hyun et al., 2021). Prolonged heavy drinking can accelerate the progression of fatty liver to hepatitis. Despite extensive studies over the years, effective treatments for alcoholic liver disease remain elusive, with no FDA-approved medications currently available for managing ALD (Park et al., 2022).

QGLD, approved by the China National Medical Products Administration in 1999 (Identifier No. Z19991028), is composed of *Artemisia capillaris Thunb*., *Gardenia jasminoides Ellis.*, *Lonicera confusa DC.*, *Magnolia officinalis Rehd.et Wils.*and *Stephania tetrandra S.Moore* (The plant nomenclature has been verified by https://wfoplantlist.org/). QGLD has been used to treat anorexia, hypochondriac pain, weariness, fatigue, yellow urine, greasy fur, and stringy pulse caused by dampness-heat accumulates in the liver and gallbladder for over 20 years. The components in QGLD have been found to have multiple pharmacological effects. The extract of *Artemisia capillaris Thunb.* can alleviate jaundice caused by α-naphthylisothiocyanate (ANIT) in mice (Yang et al., 2023). Scoparone is one of the main active components of *Artemisia capillaris* Thunb., it has been demonstrated to have anti-inflammatory (Feng et al., 2023; Liu et al., 2020a), antioxidant (Lyu et al., 2021), mitochondrial function-improving (Jiang et al., 2022), anti-fibrotic (Gao et al., 2020), and lipid metabolism-regulating abilities (Huang et al., 2024). Geniposide is one of the active components of *Gardenia jasminoides* Ellis., it has been shown to improve metabolic-associated fatty liver disease (MAFLD) by regulating the NRF2/AMPK/mTOR pathway (Shen et al., 2020). Additionally, geniposide has been shown to mitigate liver damage induced by CCl_4_ in mice by curbing oxidative stress and lessening the hepatic inflammatory response (Yang et al., 2021), and can reduce lipid accumulation in plasma and liver by regulating FXR-mediated enterohepatic circulation of bile acids (Liu et al., 2020b). *Lonicera confuse* DC. contains active components such as chlorogenic acid and luteolin. A meta-analysis has revealed that chlorogenic acid exerts significant antioxidant and anti-inflammatory effects across a spectrum of liver ailments, including alcoholic liver disease, drug-induced liver injury, MAFLD, cholestatic liver disease, and liver fibrosis, primarily through the activation of nuclear factor erythroid 2-related factor 2 (NRF2) and inhibiting the Toll-like receptor 4 (TLR4)/nuclear factor-κB (NF-κB) signaling pathway (Xue et al., 2023). Luteolin has also been identified as possessing notable anti-inflammatory and anti-cancer activities (Conti et al., 2021; Rakoczy et al., 2023). Additionally, Magnolol and honokiol derived from *Magnolia of ficinalis* Rehd.et Wils. have also been found to have anti-inflammatory and antioxidant affects (Ming-Xin Guo et al., 2023; Lin et al., 2021; Khan et al., 2023) Tetrandrine, a bisbenzylisoquinoline alkaloid sourced from *Steohania tetrandra S. Moore*, shows a diverse array of pharmacological benefits, including anti-tumor effects achieved through the regulation of autophagy (Lima et al., 2024; Bhagya and Chandrashekar, 2022), anti-inflammatory actions by inhibiting the activation of the NLRP3 inflammasome (Song et al., 2022) and the NF-κB signaling pathway (Fang et al., 2024), as well as a decrease in reactive oxygen species (ROS) production via the induction of autophagy (Liu et al., 2022). These suggest the components of QGLD possess potential in reducing lipid accumulation and exerting antioxidant effects.

Following the intestinal digestion of dietary fats into free fatty acids (FFAs) and monoglycerides, these components are absorbed into enterocytes via specific transporters, notably fatty acid transport protein 4 (FATP4) and CD36. Within enterocytes, FFAs and monoglycerides are re-esterified into triglycerides and subsequently transported to peripheral tissues (liver, muscle, adipose) via chylomicrons (Feingold, 2022; Huang et al., 2022). Existing researches have demonstrated that ethanol contributes to the accumulation of lipids in the liver by influencing hepatic lipid uptake, stimulating lipogenesis, hindering fatty acid oxidation, and affecting lipid export from the liver (Jeon and Carr, 2020). Notably, alcohol also modulates intestinal nutrient absorption by altering transporter protein activity (Butts et al., 2023). Importantly, its disruptive effects on enterohepatic lipid transport further exacerbate alcohol-associated liver pathology.

SOD is the main antioxidant enzyme that clears superoxide anion radicals, converting them into hydrogen peroxide. GSH catalyzes the decomposition of hydrogen peroxide and plays a crucial role in the body’s antioxidant defense system (Georgiou-Siafis and Tsiftsoglou, 2023). MDA, the final product of intracellular polyunsaturated fatty acid peroxidation, is a reliable biomarker for tissue lipid peroxidation processes (Chumroenvidhayakul et al., 2023). The NRF2/KEAP1 pathway is one of the main cellular defense mechanisms in response to oxidative stress (Zhang et al., 2023). NRF2 activates cellular antioxidants and induces transcription of NQO1 and HO-1 in response to oxidative stress, while KEAP1 negatively regulates NRF2 function through degradation. Studies have shown that alcohol-induced liver toxicity is significantly increased in NRF2 knockout mice (Yang et al., 2024), and other studies have found that NRF2 activity is significantly inhibited in mice after alcohol exposure (Kim et al., 2023).

There is no relevant research on whether QGLD can be used for ALD. In this paper, the acute alcohol-induced liver injury model and chronic and binge ethanol feeding model (NIAAA) were established to evaluate the efficacy of QGLD in mice. Molecular biology methods combining network pharmacology were performed to enhance understanding of the mechanisms.

## 2 Materials and Methods

### 2.1 QingGan lidan capsule

The QGLD (Lot No: 2310001) was produced by Jiangxi Conba Pharmaceutical Co., Ltd. In brief, Artemisia capillaris Thunb. (2140 g), Lonicera confusa DC. (1430 g), Gardenia jasminoides Ellis. (357.5 g), Magnolia officinalis Rehd.et Wils. (357.5 g), and Stephania tetrandra S.Moore (715 g) are decocted with water and filtered twice. The filtrates are combined, ethanol is added to achieve an ethanol concentration of 70%, and the mixture is left to stand for 24 h before filtering again. The filtered solution is concentrated and subjected to spray drying. The dry extract is mixed with starch and pulverized, then encapsulated into 1,000 capsules. The production process and quality standards of QGLD have been detailed in the 2020 edition of the Pharmacopoeia of the People’s Republic of China (Pharmacopoeia, 2020).

### 2.2 Animals

The experimental mice (C57BL/6J, male, eight weeks-old) were purchased from Zhejiang Vital River Laboratory Animal Technology Co., Ltd. All mice were kept in an environment maintained at a stable temperature of 24°C ± 2°C and a relative humidity of approximately 50%, with unrestricted access to food and water. The care of the animals adhered rigorously to the international guidelines for the ethics and welfare of experimental animals (ethics number: IACUC/HTYJ-9305-01).

### 2.3 Animal experiment design

Acute Alcoholic Liver Injury Model: 50 mice were randomly divided into five groups, including the control group (Con), model group (Model), low-dose QGLD group (QGLD-L, 360 mg/kg), medium-dose QGLD group (QGLD-M, 720 mg/kg), and high-dose QGLD group (QGLD-H, 1,440 mg/kg). The Con and Model groups received a 0.5% carboxymethyl cellulose sodium (CMC-Na) solution through gavage, whereas the other groups administered respective doses of QGLD mixed with 0.5% CMC-Na solution. After 7 days of treatment, all groups received 50% ethanol by gavage three times, while the control group was gavaged with saline. The dosage was 12 mL/kg per time, with an interval of 12 h between each dose. Blood, liver, and ileum were sampled 6 hours following the last alcohol administration.

NIAAA model: 75 mice were randomly divided into five groups with the same group settings as above. The NIAAA model was established using the commercial Lieber-DeCarli diet (Cat No.710260, Dyets, United States) (Lamas-Paz et al., 2018; Bertola et al., 2013; Gao et al., 2017). QGLD administration began when the diet alcohol concentration reached 4%, and continued for 28 days. Blood, liver and ileum were sampled 6 h after the final alcohol gavage.

The increase in liver index, serum AST, and ALT levels in the model group indicates that the model has been successfully established.

### 2.4 Determination of liver function biochemical indicators

Serum was separated from blood by centrifugation. Liver tissue was homogenized with PBS (1:10, w/v) by grinder (Jingxin, China) at 4°C, 60 Hz, for 90 s, repeated three times. After homogenization, the homogenate was centrifuged (5,000×g, 4°C, 10 min) to isolate the supernatant. Protein concentrations in the supernatant were then quantified using a BCA assay kit (Cat No.E-BC-K318-M, Elabscience, China). Serum levels of Total cholesterol (TC), Triglyceride (TG), Aspartate aminotransferase (AST), and Alanine aminotransferase (ALT) were detected following the manufacturer’s instructions. The TC and TG levels in the liver tissue homogenate were also detected.

The TC (Cat No.A111-1-1), TG (Cat No.A110-1-1), AST (Cat No.C010-2-1), and ALT (Cat No.C009-2-1) detection kits were purchased from Nanjing Jiancheng Bioengineering Institute.

### 2.5 Determination of antioxidant enzyme activity

Liver tissue homogenates were prepared as above. The levels of reduced glutathione (GSH, Cat No.A006-two to one, Nanjing Jiancheng Bioengineering Institute, China), Superoxide Dismutase (SOD, Cat No.A001-three to two, Nanjing Jiancheng Bioengineering Institute, China), and Malondialdehyde (MDA, Cat No.S0131S, Beyotime, China) in the liver tissue homogenate supernatant were detected following the manufacturer’s instructions.

### 2.6 Histological staining and analyzing

For H&E staining, samples of liver and ileum were fixed in a 4% paraformaldehyde solution. Following fixation, the samples underwent dehydration in a graded ethanol series, cleared in xylene, and embedded in paraffin at 56°C. 5-μm-thick sections were cut from the embedded samples. The slices were immersed in hematoxylin solution for 2 min, rinsed in water for 1 min, and subsequently immersed in eosin solution for 10 min. Then, the sections were differentiated in 1% hydrochloric acid-ethanol for 10 s.

For Oil Red O staining, 6-μm-thick sections were prepared. After washing off the embedding medium, the slices were washed in 60% isopropanol for 2 min, then stained with Oil Red O working solution for 2–5 min. Then the sections were differentiated in 60% isopropanol, and washed in water until the nuclei turned blue (5–10 min). The excess water was blotted, and the sections were mounted using glycerin gelatin. The above staining procedures and result images were provided by Shanghai Ruiyu Biotechnology Co., Ltd.

Quantitative analysis of Oil Red O staining: Under ×200 magnification, seven random areas were photographed. The area of Oil Red O-positive staining and the total cell coverage area were quantified by ImageJ software. The ratio between the two areas was calculated to determine the Oil Red O-stained area percentage.

Measurement of ileal villus length and spacing: The length of the 8–10 longest villi from each section and the width of 8–10 uniform villus spaces were measured by section viewing software (K-Viewer, KF BIO).

### 2.7 Quantitative real-time polymerase chain reaction (qRT-PCR)

About 20–30 mg of mouse liver or ileum samples were collected, two grinding beads and 1,000 μL of RNAiso Plus reagent (Cat No.9109, TaKaRa, Japan) were added. The samples were then homogenized in a grinder under 4°C conditions (60 Hz, 90 s, 3 repeats). Subsequently, the RNA was extracted following the protocol provided by the RNAiso Plus reagent kit. The concentration and purity of the obtained RNA were measured by a microplate reader (BioTek Synergy H1, Agilent, USA). cDNA was prepared from 1,000 ng of RNA samples employing the FastKing cDNA First-Strand Synthesis Kit (Cat No.KR116, TIANGEN Biotech, China). For the analysis of mRNA expression levels about lipid metabolism genes (PPAR-α, FASN, Srebp-1c, ACC1), oxidative stress genes (NRF2, Keap1, SOD2, CAT, HO-1, NQO1), and lipid transport genes (CD36, FABP, FATP, SR-B1, LDLR, ABCA1) in both the liver and ileum, qRT-PCR was conducted using the SuperReal PreMix Color (SYBR Green) kit. The housekeeping gene GAPDH served as the internal reference. The comparative threshold cycle (CT) method was utilized for analyzing the PCR data, and the 2^−ΔΔCt^ formula was applied. Primer sequences are listed in Supplementary Table S1.

### 2.8 Network pharmacology

Network pharmacology is a method for predicting active ingredients and elucidating the pharmacological mechanisms of compound traditional Chinese medicine (Zhao et al., 2023). Potential compounds of five herbal ingredients in QGLD were gathered from TCMSP (OB ≥ 30% and DL ≥ 0.18), and potential targets were analyzed using Swiss Target Prediction. The active ingredients associated with the expected targets were imported into the UniProt database. Targets related to alcoholic liver diseases were retrieved using the Genecards database, OMIM database, Comparative Toxicogenomics database, and NCBI. Gene symbols were confirmed using the UniProt database or accuracy. Common targets between QGLD and ALD were identified by Venny 2.1.0. KEGG and GO enrichment analyses were performed using the DAVID database and Metascape. The protein-protein interaction (PPI) network of potential targets was constructed utilizing Cytoscape with the String database.

### 2.9 Statistical analysis

Student’s t-test was employed to assess the differences between groups. Results are expressed as mean ± SEM. *p* < 0.05 was deemed statistically significant. SPSS 26.0, ImageJ, GraphPad Prism 8.0, Microsoft Excel, and Microsoft PowerPoint were used for data analysis and graph plotting.

## 3 Results

### 3.1 QGLD reduced liver ALT, AST, TC, and TG levels in acute alcoholic liver injury model

**FIGURE 1:**
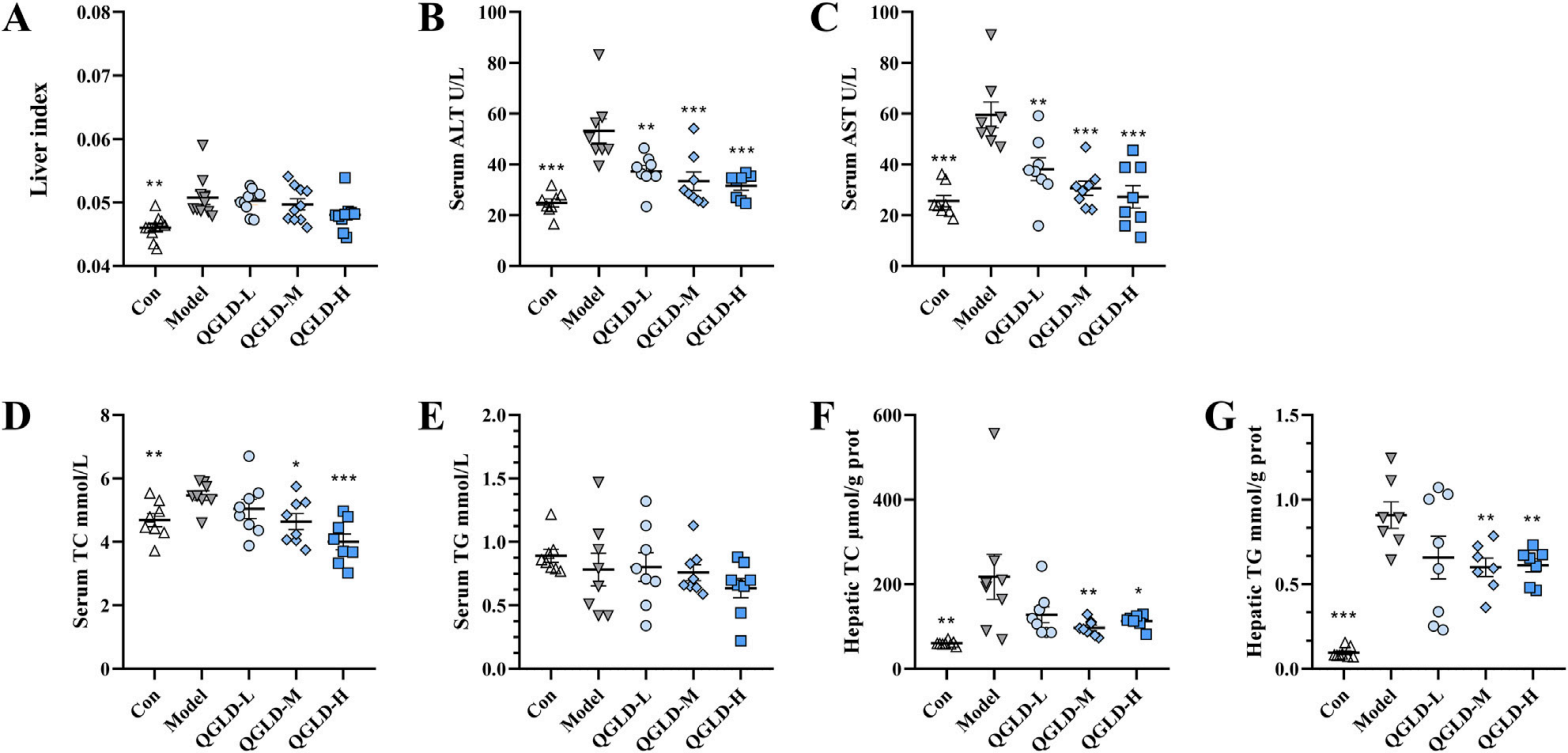
Effects of QGLD on liver ALT, AST, TC, and TG Levels in acute alcoholic liver injury model. **(A)** Liver index of mice (n = 10). **(B)** Serum ALT level (n = 8). **(C)** Serum AST level (n = 8). **(D)** Serum TC level (n = 8). **(E)** Serum TG level (n = 8). **(F)** Hepatic TC level (n = 8). **(G)** Hepatic TG level (n = 8). *p<0.05, **p<0.01 vs. the Model group.

After being exposed to 50% ethanol three times, the mice showed a significantly increased liver index. Administration of QGLD did not affect the liver index (Figure 1A). The serum ALT and AST levels were markedly elevated in the Model group. In contrast, they showed a significant reduction in the QGLD-L, QGLD-M, and QGLD-H groups (Figures 1B, C). In the Model group, acute alcohol intake significantly increased serum and liver TC levels. However, the QGLD-L group exhibited a significant decrease in liver TC levels (Figure 1D), and the QGLD-M and QGLD-H groups presented a marked reduction in both serum and liver TC levels (Figures 1D, F). Although serum TG levels in the Model group remained unchanged, liver TG levels increased significantly. In contrast, the QGLD-M and QGLD-H groups displayed significantly lower liver TG levels when compared to the Model group (Figure 1G). These findings suggest that QGLD effectively mitigates alcohol-induced hepatic lipid accumulation and regulates lipid transport.

### 3.2 QGLD reduced hepatic lipid accumulation in acute alcoholic liver injury model

**FIGURE 2:**
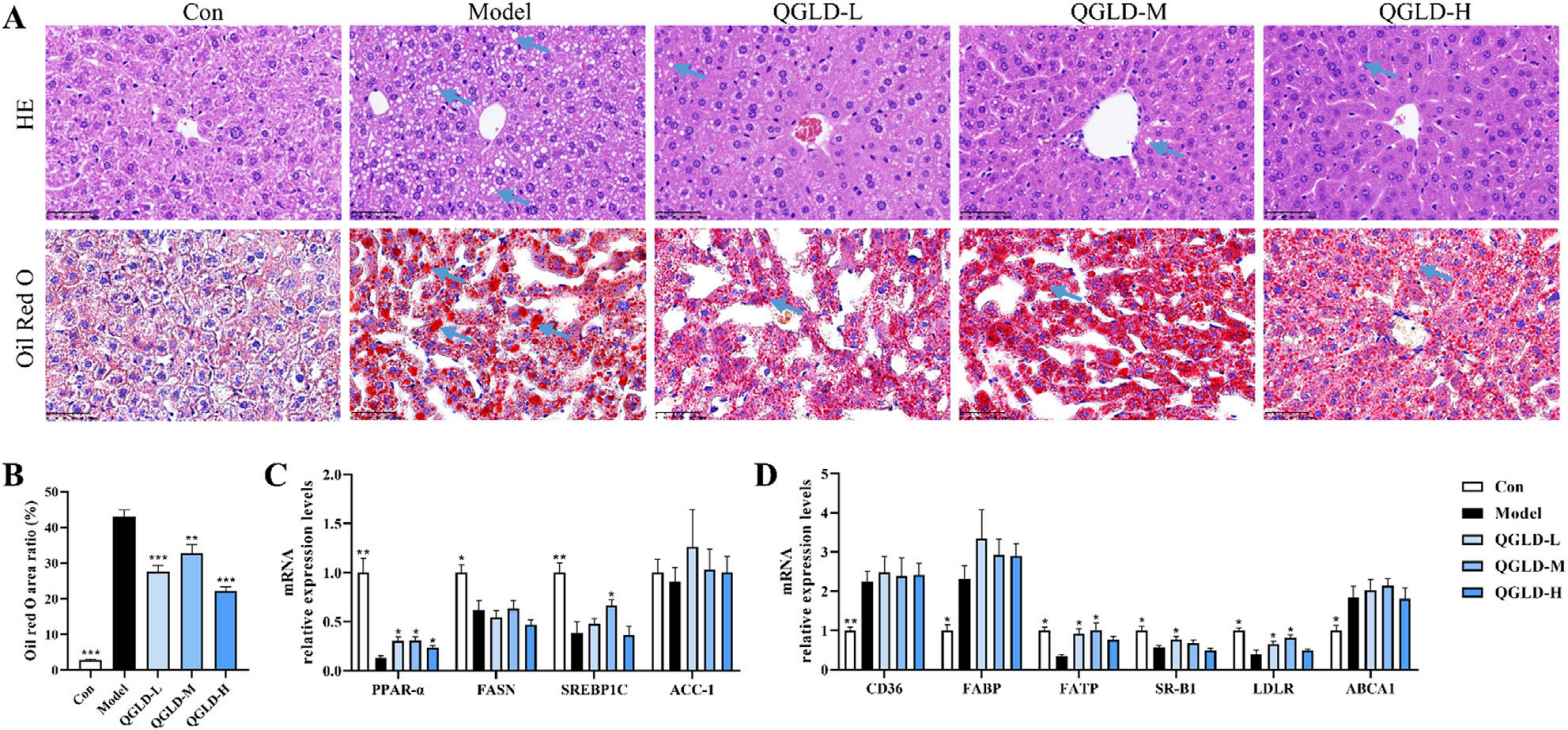
Effects of QGLD on hepatic lipid metabolism in acute alcoholic liver injury model **(A)** Representative H&E and Oil Red O staining images of the liver (Blue arrow: Lipid drop). **(B)** Quantification of the Oil Red O positive area (n = 4). **(C)** Relative mRNA expression levels of PPAR-α, FASN, SREBP1C, ACC-1 in the liver (n = 8). **(D)** Relative mRNA expression levels of CD36, FABP, FATP, SR-B1, LDLR, ABCA1 in the liver (n = 8). *p<0.05, **p<0.01 vs. the Model group.

Histological analysis via H&E staining showed regular liver cell arrangement and clear structure in the Con group (Figure 2A). After acute alcohol exposure, there was no obvious inflammation, but significant lipid droplet accumulation in the liver (Figure 2A). In contrast, QGLD treatment resulted in a marked decrease in the accumulation of lipid droplets. According to the results of Oil red O staining, the Model group exhibited a significantly larger area covered by lipid droplets when compared to the Con group, but QGLD treatment led to a considerable reduction (Figures 2A, B), which was consistent with the observed alterations in liver TG levels (Figure 1G). Further detection of the mRNA expression levels of genes associated with lipid metabolism showed that the mRNA levels of PPAR-α, FASN, and SREBP1C were significantly downregulated in the Model group relative to the control cohort. After QGLD treatment, PPAR-α expression showed a marked increase. Similarly, SREBP1C expression showed a significant increase in the QGLD-M group (Figure 2C). PPAR-α is a transcription factor that governs a multitude of genes involved in the processes of lipid synthesis, oxidation, and transport (Sun et al., 2024), suggesting the potential of QGLD to regulate lipid metabolism. Additionally, the detection of the mRNA expression levels of genes associated with lipid transport showed that the mRNA levels of CD36, FABP, and ABCA1 were notably elevated in the Model group, while those of FATP, SR-B1, and LDLR were significantly decreased. Conversely, in the QGLD-L group, the levels of FATP, SR-B1, and LDLR were increased considerably compared to the Model group, with FATP and LDLR also showing significant upregulation in the QGLD-M group (Figure 2D). FATP is a carrier for TG transport (He et al., 2023), and SR-B1 and LDLR are mainly involved in TC transport (Hoebinger et al., 2023; Fung et al., 2024). These indicated that QGLD might regulate hepatic TC and TG homeostasis by modulating lipid transport.

### 3.3 QGLD restored the antioxidant abilities in acute alcoholic liver injury model

**FIGURE 3:**
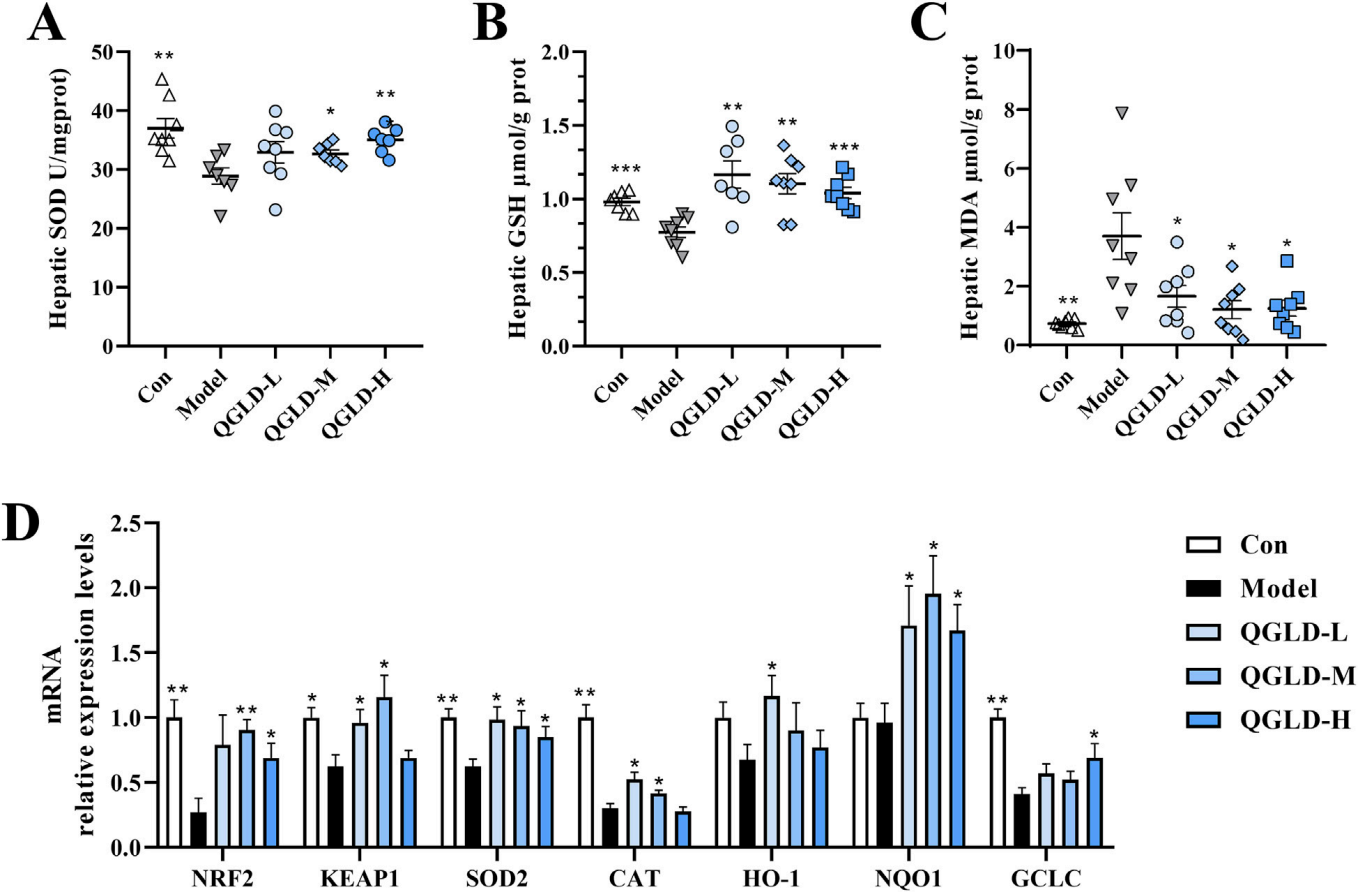
Effects of QGLD on hepatic oxidative stress in acute alcoholic liver injury model **(A)** Hepatic SOD activity (n = 8). **(B)** Hepatic GSH level (n = 8). **(C)** Hepatic MDA level (n = 8). **(D)** Relative mRNA expression levels of NRF2, KEAP1, SOD2, CAT, HO-1, NQO1 in the liver (n = 8). *p<0.05, **p<0.01 vs. the Model group.

After acute alcohol intake, the liver SOD and GSH levels in the Model group showed a notable decrease (Figures 3A, B), indicating oxidative damage to the liver. However, after QGLD treatment, the liver GSH level was significantly increased. Additionally, the liver SOD level in the QGLD-H group (Figures 3A, B), demonstrating the capacity of QGLD to enhance the activities of antioxidant enzymes in mice liver. Furthermore, the liver MDA level was significantly elevated in the Model group, but was significantly reduced by QGLD treatment (Figure 3C), suggesting that QGLD could improve alcohol-induced lipid peroxidation. Further detection of the mRNA expression levels of genes associated with oxidative stress revealed that the mRNA levels of NRF2, KEAP1, SOD2, CAT and GCLC were significantly downregulated in the Model group relative to the control cohort. In contrast, the mRNA levels of KEAP1, SOD2, CAT, and HO-1 in the QGLD-L group were significantly increased relative to the Model group. Meanwhile, the mRNA levels of NRF2, KEAP1, SOD2, CAT and NQO1 were increased considerably in the QGLD-M group. The mRNA levels of NRF2, SOD2, NQO1 and GCLC were significantly increased in the QGLD-H group (Figure 3D). These findings suggested that QGLD may restore the antioxidant capacity through transcriptional regulation of the NRF2/KEAP1 pathway.

### 3.4 QGLD affected the lipid transport of ileum in acute alcoholic liver injury model

**FIGURE 4:**
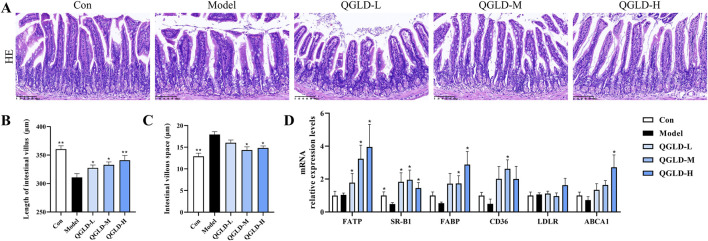
Effect of QGLD on ileum in acute alcoholic liver injury model **(A)** Representative H&E staining images of the ileum. **(B)** Quantification of the intestinal villus (n = 4). **(C)** Quantification of the villous space (n = 4). **(D)** Relative mRNA expression levels of NRF2, KEAP1, SOD2, CAT, HO-1, NQO1 in the liver (n = 8). *p<0.05, **p<0.01 vs. the Model group.

Histological analysis via H&E staining demonstrated that the ileal villi in the Con group exhibited normal morphology with no obvious damage to goblet cells (Figure 4A). However, the villus length in the Model group was markedly shortened, and the intervillous space was increased, indicating a decrease in ileal villus density. After QGLD treatment, the villus length and villous space in the QGLD-M and QGLD-H groups showed significant enhancement when contrasted with the Model group. These findings suggested that QGLD improved acute alcohol-induced ileal villus injury. Additionally, the expression levels of mRNA for genes associated with lipid transport in the ileum were analyzed, given the ileum’s important role in lipid absorption and metabolism. In comparison to the Con group, the Model group exhibited a significant decrease in the mRNA levels of SR-B1 (Figure 4D). However, the mRNA levels of SR-B1 in the QGLD-L group increased significantly relative to the model group; the mRNA levels of FATP, SR-B1, FABP, and CD36 in the QGLD-M group increased significantly relative to the model group; the mRNA levels of FATP, SR-B1, and FABP in the QGLD-H group increased significantly relative to the model group (Figure 4D). These findings implied that QGLD might regulate hepatic lipid accumulation by affecting lipid transport.

### 3.5 QGLD reduced liver ALT, AST, TC, and TG levels in NIAAA model

**FIGURE 5:**
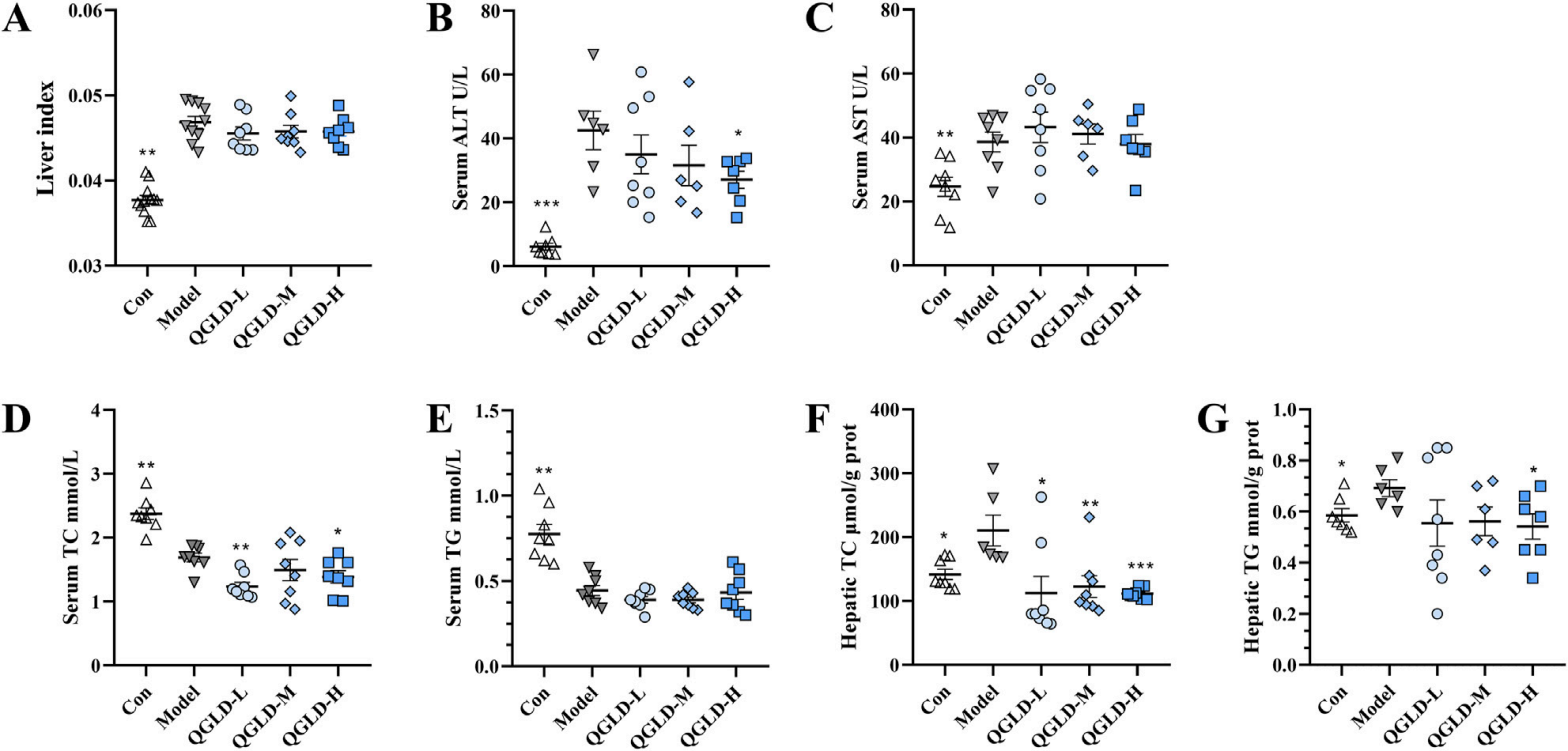
Effects of QGLD on liver ALT, AST, TC, and TG Levels in NIAAA model **(A)** Liver index of mice (n = 8). **(B)** Serum ALT level (n = 8). **(C)** Serum AST level (n = 8). **(D)** Serum TC level (n = 8). **(E)** Serum TG level (n = 8). **(F)** Hepatic TC level (n = 8). **(G)** Hepatic TG level (n = 8). *p<0.05, **p<0.01 vs. the Model group.

The Model group showed a notable increase in the liver index. However, treatment with QGLD did not seem to have any effect on this parameter (Figure 5A). Serum ALT and AST levels were markedly increased in the Model group, while the ALT levels in the QGLD-H group were markedly downregulated (Figure 5B), whereas AST levels remained relatively unchanged after QGLD treatment (Figure 5C). Furthermore, the serum TC levels in the Model group showed a significant decrease, while liver TC levels exhibited a significant increase (Figures 5D, F). Compared to the Model group, the serum TC level in the QGLD-L and QGLD-H groups decreased significantly (Fig. 5D), and the hepatic TC level decreased significantly after QGLD treatment (Figure 5F). There was no significant change in the serum TG levels after QGLD treatment. However, the liver TG level in the Model group increased significantly (Figure 5G). The liver TG level in the QGLD-H group exhibited a significant decrease compared to the Model group (Figure 5G). These findings indicated that QGLD can mitigate alcohol-induced liver lipid accumulation.

### 3.6 QGLD reduced hepatic lipid accumulation in NIAAA model

**FIGURE 6:**
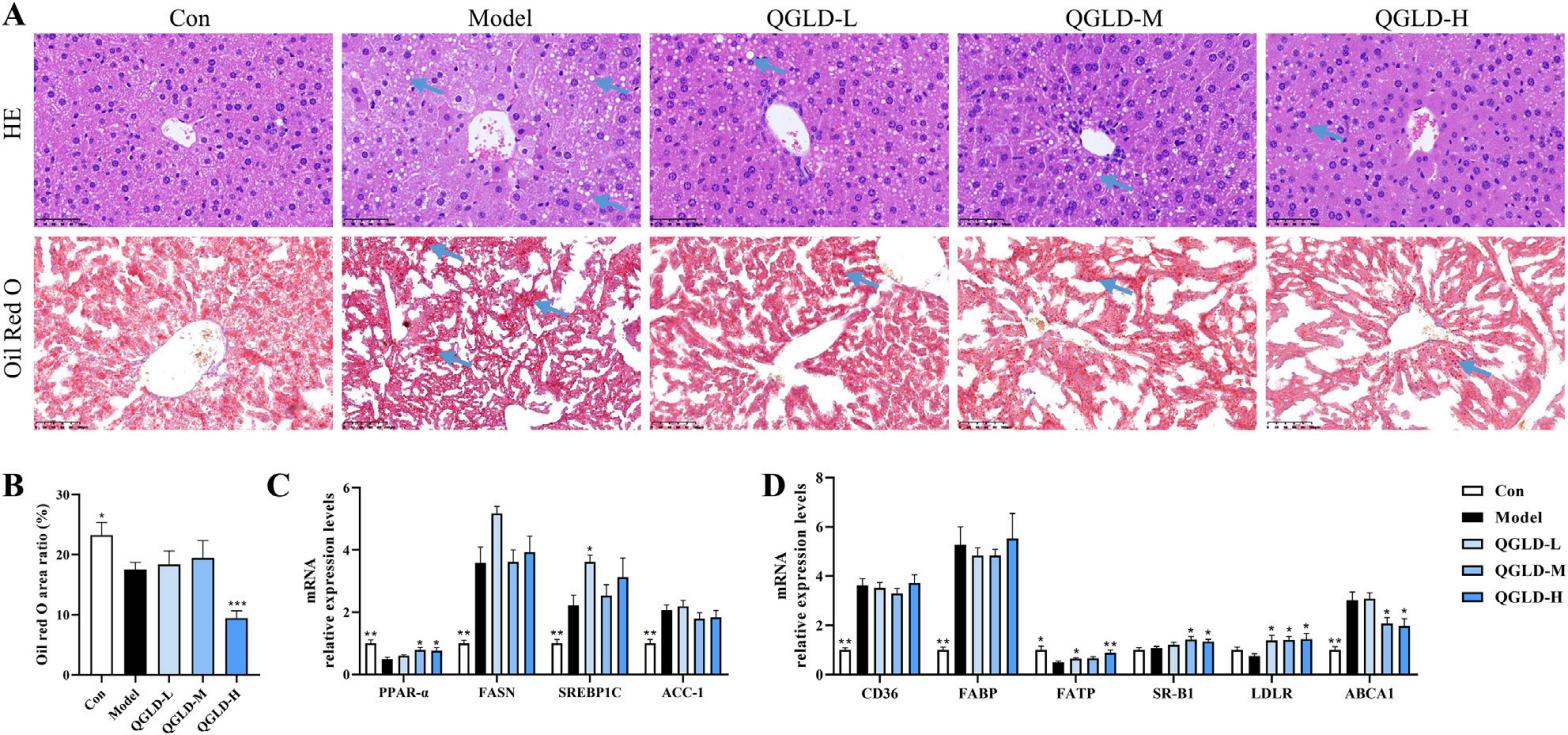
Effects of QGLD on hepatic lipid metabolism in NIAAA model. **(A)** Representative H&E and Oil Red O staining images of the liver (Blue arrow: Lipid drop). **(B)** Quantification of the Oil Red O positive area (n = 4). **(C)** Relative mRNA expression levels of PPAR-α, FASN, SREBP1C, ACC-1 in the liver (n = 8). **(D)** Relative mRNA expression levels of CD36, FABP, FATP, SR-B1, LDLR, ABCA1 in the liver (n = 8). *p<0.05, **p<0.01 vs. the Model group.

Histological analysis via H&E staining demonstrated that the liver cells of the Model group exhibited mild swelling and obvious lipid droplet accumulation, but no evident inflammatory infiltration was observed. After the administration of QGLD, the accumulation of lipid droplets was significantly reduced (Figure 6A). Oil Red O staining reinforced this observation, showing a significantly lower area of lipid droplet coverage in the QGLD-H group relative to the Model group (Figure 6A), which aligned with the observed changes in liver TG levels (Figure 5G). Interestingly, the lipid droplet coverage in the Model group was lower than that in the Con group, which may be due to the aversion of mice to alcohol, leading to changes in food intake (Gao et al., 2017). These findings suggested that QGLD can reduce the liver lipid accumulation induced in the NIAAA model. Further analysis of mRNA expression levels related to lipid metabolism and transport revealed that, compared to the Con group, the levels of PPAR-α mRNA were notably decreased in the Model group, while FASN, SREBP1C, and ACC-1 mRNA levels were significantly increased (Figure 6C), potentially contributing to liver lipid accumulation. In contrast, the mRNA levels of PPAR-α were markedly elevated in the QGLD-M and QGLD-H groups, aligning with observations in the acute alcoholic liver injury model, which indicated that QGLD has the potential to regulate lipid metabolism and transport. Compared to the Con group, the mRNA levels of CD36, FABP, and ABCA1 were significantly upregulated, while the mRNA level of FATP was significantly downregulated in the Model group. Compared to the Model group, the mRNA levels of FATP and LDLR were significantly upregulated in the QGLD-L group, the mRNA levels of SR-B1 and LDLR were significantly upregulated in the QGLD-M group, and the mRNA levels of FATP, SR-B1, and LDLR were significantly upregulated in the QGLD-H group. The mRNA levels of ABCA1 were significantly downregulated in the QGLD-M and QGLD-H groups (Figure 6D). These findings indicated that QGLD may affect the homeostasis of liver TC and TG by regulating lipid transport.

### 3.7 QGLD restored the antioxidant abilities in NIAAA model

**FIGURE 7:**
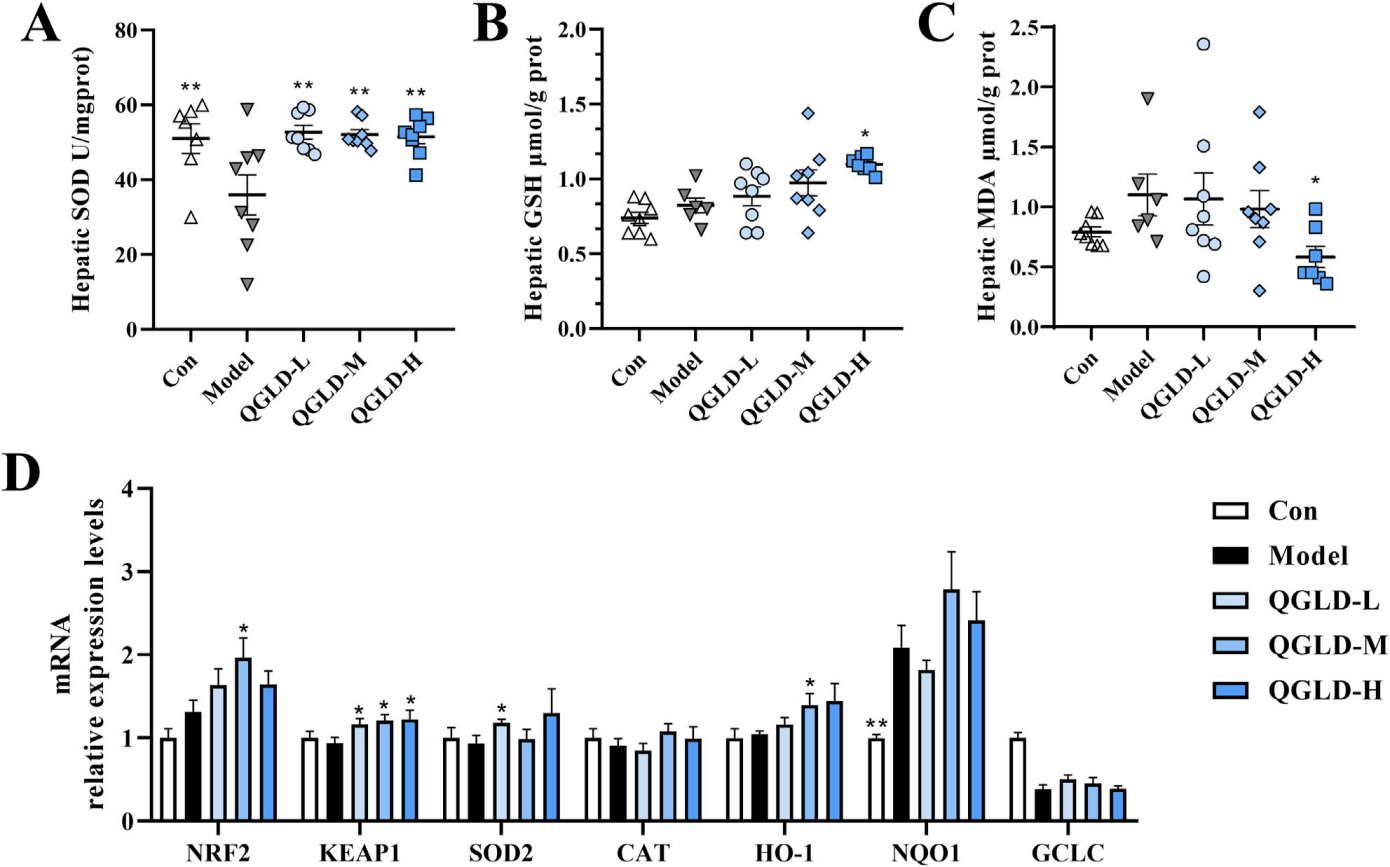
Effects of QGLD on hepatic oxidative stress in NIAAA model. **(A)** Hepatic SOD activity (n = 8). **(B)** Hepatic GSH level (n = 8). **(C)** Hepatic MDA level (n = 8). **(D)** Relative mRNA expression levels of NRF2, KEAP1, SOD2, CAT, HO-1, NQO1 in the liver (n = 8). *p<0.05, **p<0.01 vs. the Model group.

After NIAAA modeling, the liver SOD levels of the Model group were markedly reduced. In contrast, the QGLD treatment led to a notable increase in liver SOD levels (Figure 7A). The GSH levels of the Model group showed no significant change compared to the Con group. In contrast, the GSH levels in the QGLD-H group were significantly elevated compared to the Model group (Figure 7B). Additionally, the MDA level of the QGLD-H group was significantly lower than that in the Model group (Figure 7C). These results indicated that high-dose QGLD can enhance the antioxidant capacity of the liver in mice.

**FIGURE 8:**
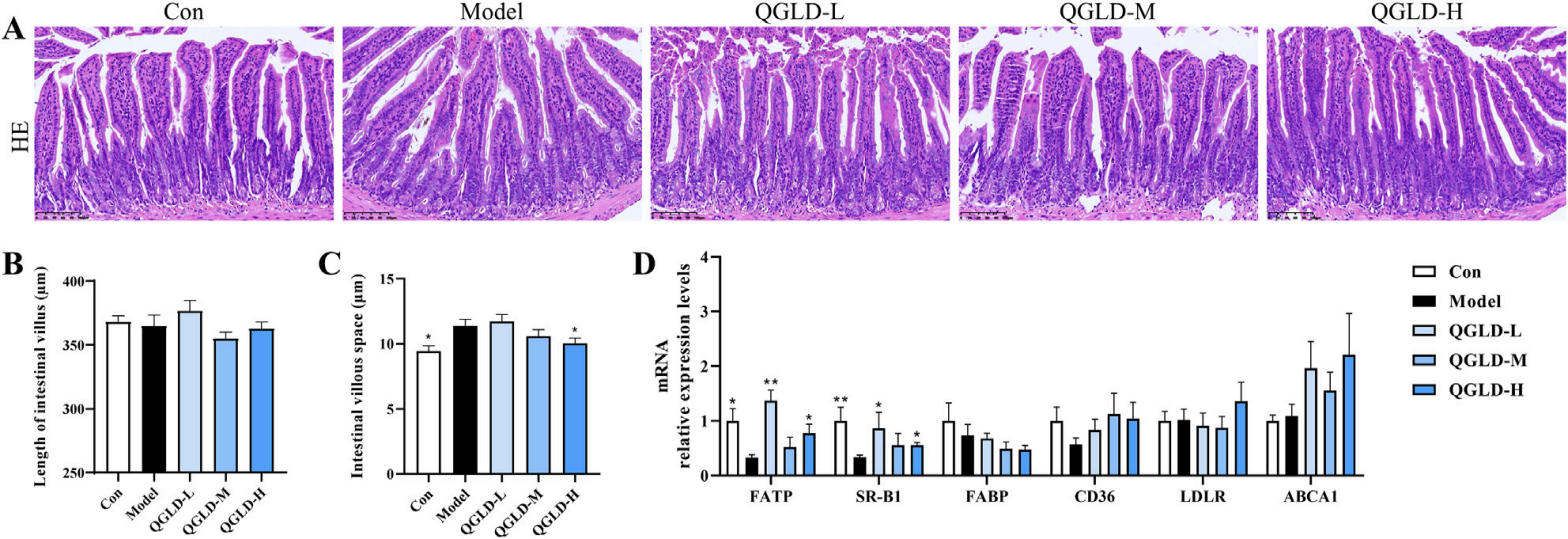
Effect of QGLD on ileum in NIAAA model. **(A)** Representative H&E staining images of the ileum. **(B)** Quantification of the intestinal villus (n = 4). **(C)** Quantification of the villous space (n = 4). **(D)** Relative mRNA expression levels of NRF2, KEAP1, SOD2, CAT, HO-1, NQO1 in the liver (n = 8). *p<0.05, **p<0.01 vs. the Model group.

The mRNA expression levels of genes related to oxidative stress were further analyzed. The result indicated that there was no notable difference between the Model group and the Con group (Figure7D). In our study, compared to the Model group, the mRNA levels of KEAP1 and SOD2 in the QGLD-L group increased significantly; the mRNA levels of NRF2, KEAP1, and HO-1 in the QGLD-M group increased significantly; the mRNA level of KEAP1 in the QGLD-H group increased significantly (Figure 8D). Although no significant difference existed between the Model group and the Con group, QGLD upregulated the mRNA levels of NRF2, KEAP1, SOD2, and NQO1, consistent with findings in the acute alcoholic liver injury model, indicating that QGLD could regulate oxidative stress.

### 3.8 QGLD affected the lipid transport of ileum in NIAAA model

Histological analysis via H&E staining demonstrated that the ileal villi in the Con group exhibited normal morphology with no apparent damage to goblet cells (Figure 8A). No significant changes were observed in villi length between groups (Figure 8B). However, the villous space was increased in the model group (Figure 8C), indicating a reduction in ileal villi density. Notably, the villous space in the QGLD-H group was smaller than that in the model group (Figure 8C). These findings implied that the NIAAA model did not significantly harm the ileum in mice.

The mRNA expression levels of genes associated with lipid transport in the ileum were examined. There was a marked decrease in the mRNA levels of SR-B1 and FATP within the Model group (Figure 8D). In contrast, compared to the model group, the mRNA level of FATP was significantly increased in the QGLD-L group, and the mRNA levels of FATP and SR-B1 were increased considerably in the QGLD-H group (Figure 8D). The observed trends in the expression of these genes were consistent with those seen in the acute alcoholic liver injury model. Therefore, QGLD might also regulate the hepatic lipid homeostasis in NIAAA model by affecting the lipid homeostasis in the liver and ileum.

### 3.9 Network pharmacology analysis results

**FIGURE 9:**
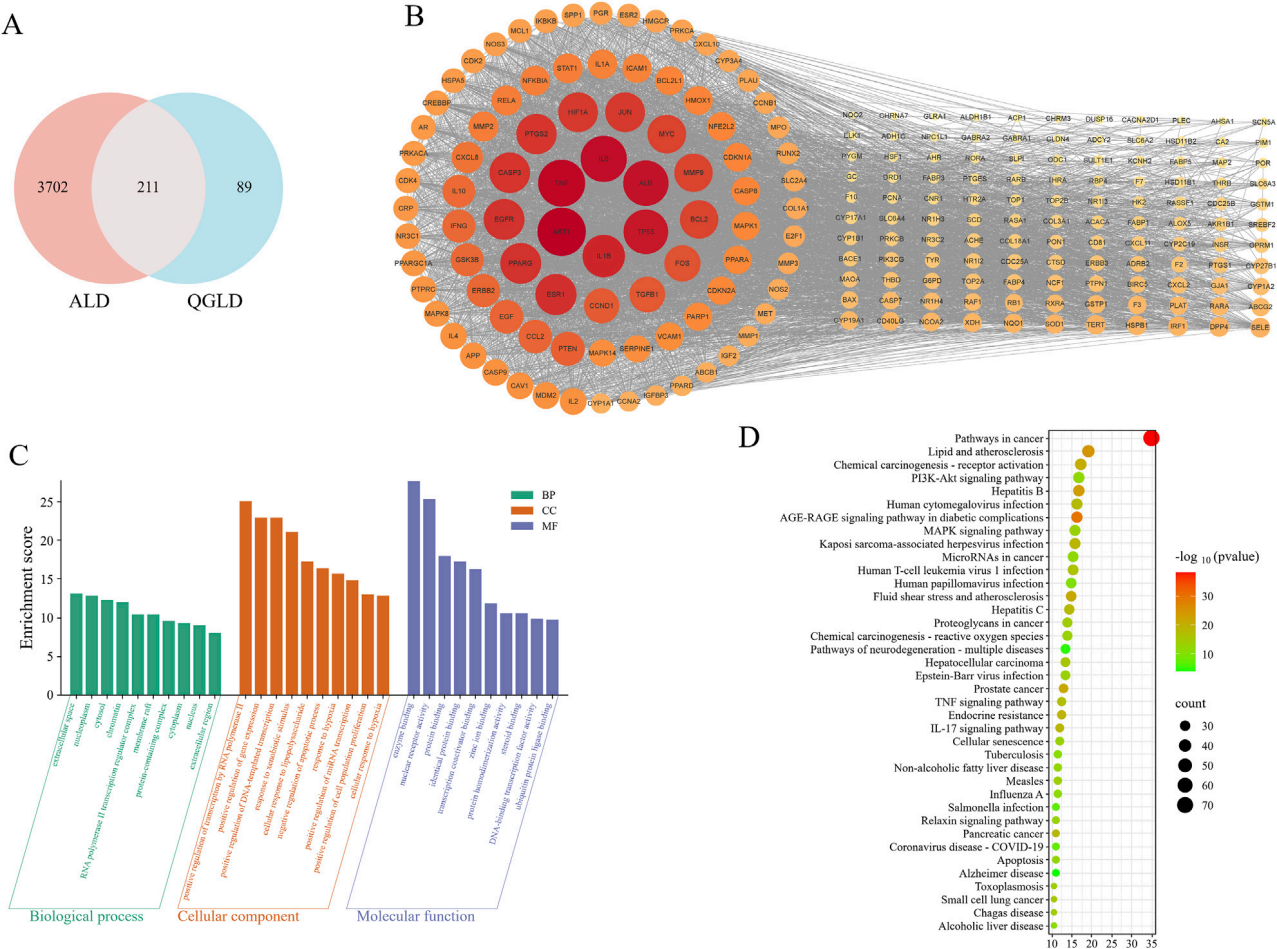
Network pharmacology analysis results **(A)** Venn diagram between target genes. **(B)** PPI network constructed using target genes, the redder the color of the dot and the larger the dot, the higher the degree value. **(C)** The top 10 GO terms were identified based on *p* values <0.01. **(D)** The top 38 pathways were identified based on *p* values <0.01.

Thirty-seven active ingredients were identified using the TCMSP database (Supplementary Table S3). Three hundred potential targets of active components were predicted using the TCMSP and Swiss Target Prediction databases. Additionally, 3913 targets related to ALD were retrieved from the GeneCards, OMIM, Comparative Toxicogenomics, and NCBI databases. Two hundred and twenty-one potential QGLD targets associated with ALD were identified by Venn diagram (Figure 9A). A protein-protein interaction (PPI) network was constructed using the STRING database, pinpointing key targets such as AKT1, TNF, IL6, ALB, TP53, and IL1B (Figure 9B). These findings suggested that QGLD may have the potential to regulate inflammation in ALD.

KEGG and GO enrichment analyses were performed utilizing the Metascape and DAVID databases. The GO analysis results highlighted pathways such as “response to xenobiotic stimulus,” “cellular response to lipopolysaccharide,” and “cellular response to hypoxia”, reflecting the cellular capacity to respond to environmental and stress-related changes. Furthermore, terms like “nuclear receptor activity” and “transcription coactivator binding” underscored the regulatory roles of proteins involved in gene expression (Figure 9C). KEGG analysis identified 178 pathways. The 38 pathways involving the most targets were displayed in Figure 9D. “Lipid and atherosclerosis” and “Non-alcoholic fatty liver disease” pathways indicated the potential of QGLD to regulate lipid metabolism, “Chemical carcinogenesis - reactive oxygen species” suggested the potential in modulating oxidative stress. Additionally, “Hepatitis B,” “Hepatitis C,” “Hepatocellular carcinoma,” and “Alcoholic liver disease” suggested that QGLD may have therapeutic potential for various liver diseases (Figure 9D).

To preliminarily validate network pharmacology predictions, we analyzed the mRNA expression levels of several genes associated with the NF-κB, PI3K-AKT, AMPK signaling pathways, and macrophage M2 polarization markers (Supplementary Figures S1). Our data demonstrated that QGLD exerted no significant regulatory effects on genes related to the NF-κB, PI3K-AKT, or AMPK pathways. Notably, QGLD markedly downregulated MRC1 and ARG1 expressions (Supplementary Figures S1C, D), suggesting its potential role in suppressing macrophage M2 polarization.

## 4 Discussion

This study preliminarily explored the pharmacological effects of QGLD on alcohol-induced liver injury in both the acute alcoholic liver injury and NIAAA models, focusing on fatty degeneration and oxidative stress. Biochemical and pathological examinations revealed significant impairment of liver function in both models, with evident lipid accumulation and increased oxidative stress levels, common manifestations of alcoholic liver injury (Lamas-Paz et al., 2018; Xiao et al., 2023). QGLD was found to reduce the serum levels of ALT and AST, indicating improved liver function. Additionally, QGLD reduced hepatic TC and TG levels, as evidenced by H&E and Oil Red O staining, which showed reduced alcohol-induced lipid accumulation. *In vivo*, lipid transport in the liver and intestines maintains lipid homeostasis (Kimura et al., 2020). RT-qPCR results demonstrated that QGLD treatment significantly upregulated the mRNA levels of lipid metabolism and transport related genes PPAR-α, FATP, SR-B1, LDLR in the liver. Meanwhile, the mRNA levels of FATP, FABP, SR-B1 and CD36 genes in the ileum were also significantly upregulated. Therefore, it could be inferred that QGLD can reduce lipid accumulation in the liver by regulating the lipid homeostasis between the liver and ileum.

In our study, the model groups exhibited significant decreases in liver SOD and GSH levels and a significant increase in MDA levels in both models. After QGLD treatment, liver SOD and GSH levels increased, while MDA levels decreased. RT-qPCR results showed a significant decrease in the mRNA levels of NRF2, KEAP1, SOD2, and CAT in the model group, whereas these genes, along with NQO1, were upregulated following QGLD treatment. These findings indicate that alcohol disrupts the balance of the NRF2/KEAP1 pathway, and QGLD effectively promotes its recovery through transcriptional regulation.

Since 70% of alcohol absorption is located in the proximal small intestine (Yan et al., 2023), which is also the primary site for lipid absorption (Zarkada et al., 2023), we examined the condition of the villus in the ileum. According to the H&E staining results, after acute alcohol exposure, the Model group exhibited shortened intestinal villi and increased villous spacing, indicating damage to the ileal villi in mice. In contrast, no similar damage was observed in the NIAAA model. This difference may be attributed to the distinct alcohol consumption patterns between the two models. In the acute alcoholic liver injury model, the ileum is exposed to higher concentrations of alcohol over a short period. But, in the NIAAA model, mice consume low concentrations of alcohol via the Lieber-DeCarli diet. Consequently, the lower concentration and slower exposure to alcohol in the NIAAA model may have less impact on ileal villus morphology.

QGLD can ameliorate liver function impairment, reduce hepatic lipid accumulation, and lower oxidative stress levels in both acute alcoholic liver injury models and NIAAA models in mice. Additionally, it may exert liver injury protection by regulating lipid metabolism, lipid transport and oxidative stress pathways. QGLD can improve the density of ileal villi in mice after acute alcohol exposure. This provides a theoretical basis for expanding the application of QGLD in alcohol-related disorders.

## 5 Conclusion

**FIGURE 10:**
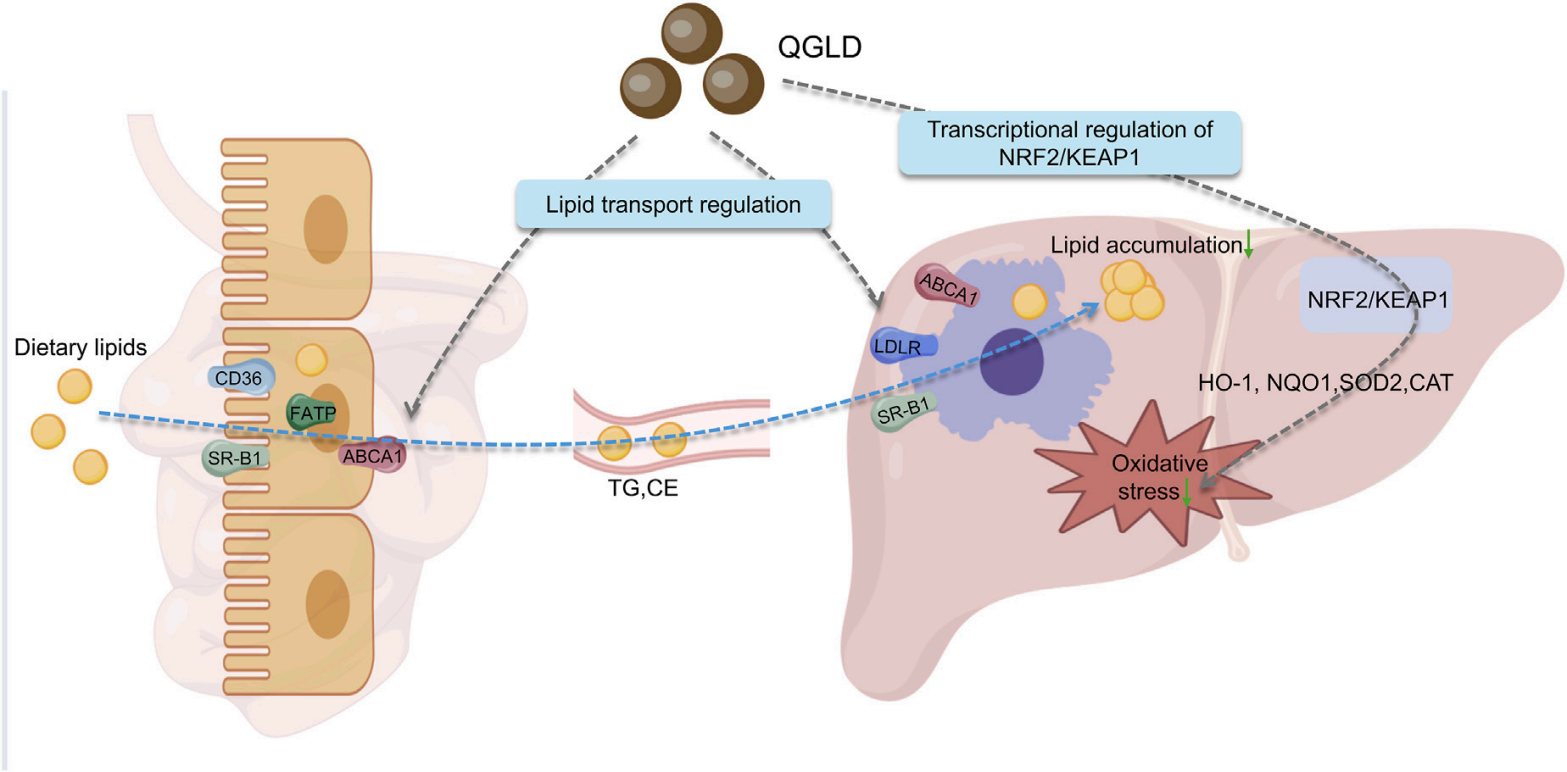
Graphical abstract of QGLD pharmacological mechanism. QGLD intervened in the process of lipid intestinal-hepatic transport by regulating the mRNA expression levels of lipid transport proteins, therefore reducing hepatic lipid accumulation. QGLD alleviated hepatic oxidative stress levels through the transcriptional regulation of NRF2/KEAP1.

QGLD, a well-established traditional Chinese medicine formulation, has demonstrated multidimensional therapeutic potential in ameliorating alcoholic liver injury through the regulation of lipid transport and oxidative stress. This study revealed the innovative mechanism by which QGLD improves alcoholic liver injury via a dual-target regulatory mode of the “gut-liver axis”: QGLD not only activates the hepatic NRF2/KEAP1 pathway to enhance antioxidant defense, but also improves lipid enterohepatic circulation by modulating lipid transport genes in both liver and intestine, thereby reducing hepatic lipid accumulation (Figure 10). The therapeutic efficacy observed in both acute and chronic ALD models suggest QGLD’s dual-stage intervention capability, providing novel insights into the multi-target therapeutic strategy of traditional Chinese medicine.

Although this study preliminarily validated QGLD’s therapeutic potential in alcoholic liver disease (ALD) models, several limitations warrant attention. The current experiments primarily focused on phenotypic improvements, while the specific molecular mechanisms underlying QGLD’s regulation of lipid transport and oxidative stress remain insufficiently explored. Future investigations should conduct in-depth analysis of its effective mechanisms and active constituents to explore broader potential applications. This research presents unique opportunities for innovation in ALD therapeutics. Through further mechanistic studies and component analysis, QGLD is expected to evolve from “drug repurposing” to a novel multi-mechanism synergistic therapeutic agent for ALD treatment.

## Data Availability

The raw data supporting the conclusions of this article will be made available by the authors, without undue reservation.
