# Supplementary figures and images for: QingGan LiDan capsules improved alcoholic liver injury by regulating liver lipid transport and oxidative stress in mice
Source: Front Pharmacol. 2025 Mar 26;16:1575280. doi: 10.3389/fphar.2025.1575280 (PMC11979125; doi:10.3389/fphar.2025.1575280)

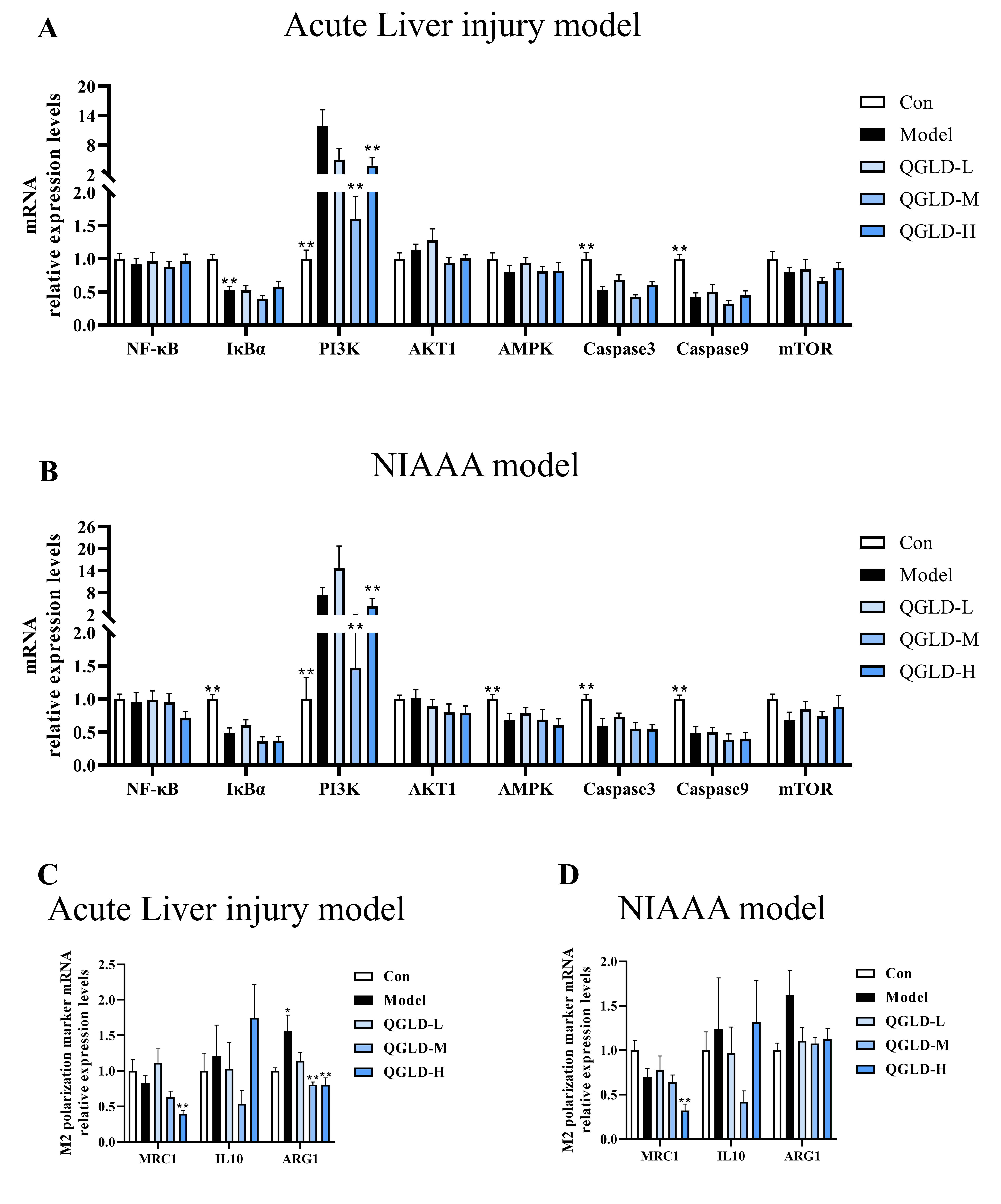

Supplement: Supplementary file 1 [file Image1.tif]
