# Supplementary material for: QingGan LiDan capsules improved alcoholic liver injury by regulating liver lipid transport and oxidative stress in mice
Source: Front Pharmacol. 2025 Mar 26;16:1575280. doi: 10.3389/fphar.2025.1575280 (PMC11979125; doi:10.3389/fphar.2025.1575280)
Supplement: Supplementary file 2 [file DataSheet1.docx]

Supplementary Material

# Supplementary Tables

**Supplementary Table 1.** The sequence of primers used in RT-qPCR

| Gene name | Primer sequence |
| --- | --- |
| *m GAPDH* | Forward 5’- AGGTCGGTGTGAACGGATTTG-3’  Reverse 5’- TGTAGACCATGTAGTTGAGGTCA-3’ |
| *m PPAR-α* | Forward 5’-AGAGCCCCATCTGTCCTCTC-3’  Reverse 5’-ACTGGTAGTCTGCAAAACCAAA-3’ |
| *m Fasn* | Forward 5’-GGAGGTGGTGATAGCCGGTAT-3’  Reverse 5’-TGGGTAATCCATAGAGCCCAG-3’ |
| *m Srebp-1c* | Forward 5’-TGACCCGGCTATTCCGTGA-3’  Reverse 5’-CTGGGCTGAGCAATACAGTTC-3’ |
| *m ACC1* | Forward 5’-GATGAACCATCTCCGTTGGC-3’  Reverse 5’-GACCCAATTATGAATCGGGAGTG-3’ |
| *m NRF2* | Forward 5’-TCTTGGAGTAAGTCGAGAAGTGT-3’  Reverse 5’-GTTGAAACTGAGCGAAAAAGGC-3’ |
| *m Keap1* | Forward 5’-TGCCCCTGTGGTCAAAGTG-3’  Reverse 5’-GGTTCGGTTACCGTCCTGC-3’ |
| *m SOD2* | Forward 5’-CAGACCTGCCTTACGACTATGG-3’  Reverse 5’-CTCGGTGGCGTTGAGATTGTT-3’ |
| *m CAT* | Forward 5’-AGCGACCAGATGAAGCAGTG-3’  Reverse 5’-TCCGCTCTCTGTCAAAGTGTG-3’ |
| *m HO-1* | Forward 5’-AAGCCGAGAATGCTGAGTTCA-3’  Reverse 5’-GCCGTGTAGATATGGTACAAGGA-3’ |
| *m NQO1* | Forward 5’-AGGATGGGAGGTACTCGAATC-3’  Reverse 5’-AGGCGTCCTTCCTTATATGCTA-3’ |
| *m CD36* | Forward 5’-ATGGGCTGTGATCGGAACTG-3’  Reverse 5’-GTCTTCCCAATAAGCATGTCTCC-3’ |
| *m FABP* | Forward 5’-AAGGTGAAGAGCATCATAACCCT-3’  Reverse 5’-TCACGCCTTTCATAACACATTCC-3’ |
| *m FATP* | Forward 5’-CGCTTTCTGCGTATCGTCTG-3’  Reverse 5’-GATGCACGGGATCGTGTCT-3’ |
| *m SR-B1* | Forward 5’-TTTGGAGTGGTAGTAAAAAGGGC-3’  Reverse 5’-TGACATCAGGGACTCAGAGTAG-3’ |
| *m LDLR* | Forward 5’-TGACTCAGACGAACAAGGCTG-3’  Reverse 5’-ATCTAGGCAATCTCGGTCTCC-3’ |
| *m ABCA1* | Forward 5’-GCTTGTTGGCCTCAGTTAAGG-3’  Reverse 5’-GTAGCTCAGGCGTACAGAGAT-3’ |

**Supplementary Table 2.** The websites of database used in network Pharmacology

| Database | Websites |
| --- | --- |
| Traditional Chinese Medicine Systems Pharmacology Database(TCMSP) | <https://old.tcmsp-e.com/> |
| Swiss Target Prediction | <http://www.swisstargetprediction.ch/> |
| UniProt database | <https://www.uniprot.org/> |
| Genecards database | <https://www.genecards.org/> |
| OMIM database | <https://www.omim.org/> |
| Comparative Toxicogenomics database | <https://ctdbase.org/> |
| NCBI | <https://www.ncbi.nlm.nih.gov/gene/> |
| Venny 2.1.0 | <https://bioinfogp.cnb.csic.es/tools/venny/> |
| DAVID database | <https://davidbioinformatics.nih.gov/tools.jsp> |
| Metascape | <https://metascape.org/> |
| String database | <https://cn.string-db.org/> |

**Supplementary Table 3.** Potential active ingredients in QGLD

| Number | Name | Pubchem Cid | CAS |
| --- | --- | --- | --- |
| 1 | 4'-Methylcapillarisin | 5319540 | 520-12-7 |
| 2 | Areapillin | 158311 | 83162-82-7 |
| 3 | beta-sitosterol | 222284 | 83-46-5 |
| 4 | capillarisin | 5281342 | 56365-38-9 |
| 5 | Demethoxycapillarisin | 5316511 | 61854-36-2 |
| 6 | Eupalitin | 5748611 | 29536-41-2 |
| 7 | Eupatolitin | 5317291 | 29536-44-5 |
| 8 | Genkwanin | 5281617 | 437-64-9 |
| 9 | Isoarcapillin | 5318508 | 85819-50-7 |
| 10 | isorhamnetin | 5281654 | 480-19-3 |
| 11 | quercetin | 5280343 | 73123-10-1 |
| 12 | Skrofulein | 188323 | 6601-62-3 |
| 13 | crocetin | 5281232 | 27876-94-4 |
| 14 | (4aS,6aR,6aS,6bR,8aR,10R,12aR,14bS)-10-hydroxy-2,2,6a,6b,9,9,12a-heptamethyl-1,3,4,5,6,6a,7,8,8a,10,11,12,13,14b-tetradecahydropicene-4a-carboxylic acid | 11869658 | 508-02-1 |
| 15 | Ammidin | 10212 | 482-44-0 |
| 16 | Sudan III | 62331 | 85-86-9 |
| 17 | kaempferol | 5280863 | 520-18-3 |
| 18 | Stigmasterol | 5280794 | 83-48-7 |
| 19 | Mandenol | 5282184 | 544-35-4 |
| 20 | Supraene | 638072 | 111-02-4 |
| 21 | isoimperatorin | 68081 | 482-45-1 |
| 22 | Ethyl oleate (NF) | 5363269 | 111-62-6 |
| 23 | 5-hydroxy-7-methoxy-2-(3,4,5-trimethoxyphenyl)chromone | 10970376 | 18103-41-8 |
| 24 | 3-Methylkempferol | 5280862 | 1592-70-7 |
| 25 | GBGB | 3082301 | 29307-60-6 |
| 26 | Ethyl linolenate | 6371716 | 1191-41-9 |
| 27 | phytofluene | 6436722 | 540-05-6 |
| 28 | Eriodyctiol (flavanone) | 373261 | 4049-38-1 |
| 29 | beta-carotene | 5280489 | 7235-40-7 |
| 30 | ZINC03978781 | 11870462 | 19716-26-8 |
| 31 | Chryseriol | 5280666 | 491-71-4 |
| 32 | kryptoxanthin | 5281235 | 472-70-8 |
| 33 | 4,5'-Retro-.beta.,.beta.-Carotene-3,3'-dione, 4',5'-didehydro- | 5380108 | 116-30-3 |
| 34 | luteolin | 5280445 | 491-70-3 |
| 35 | Eucalyptol | 34365085 | 97-53-0 |
| 36 | Tetraneurin A | 174868 | 22621-72-3 |
| 37 | Hesperetin | 72281 | 520-33-2 |

**Supplementary Fig S1.** mRNA expression levels of some genes related to NF-κB pathway, PI3K-AKT pathway, AMPK pathway, and M2 polarization markers in macrophages


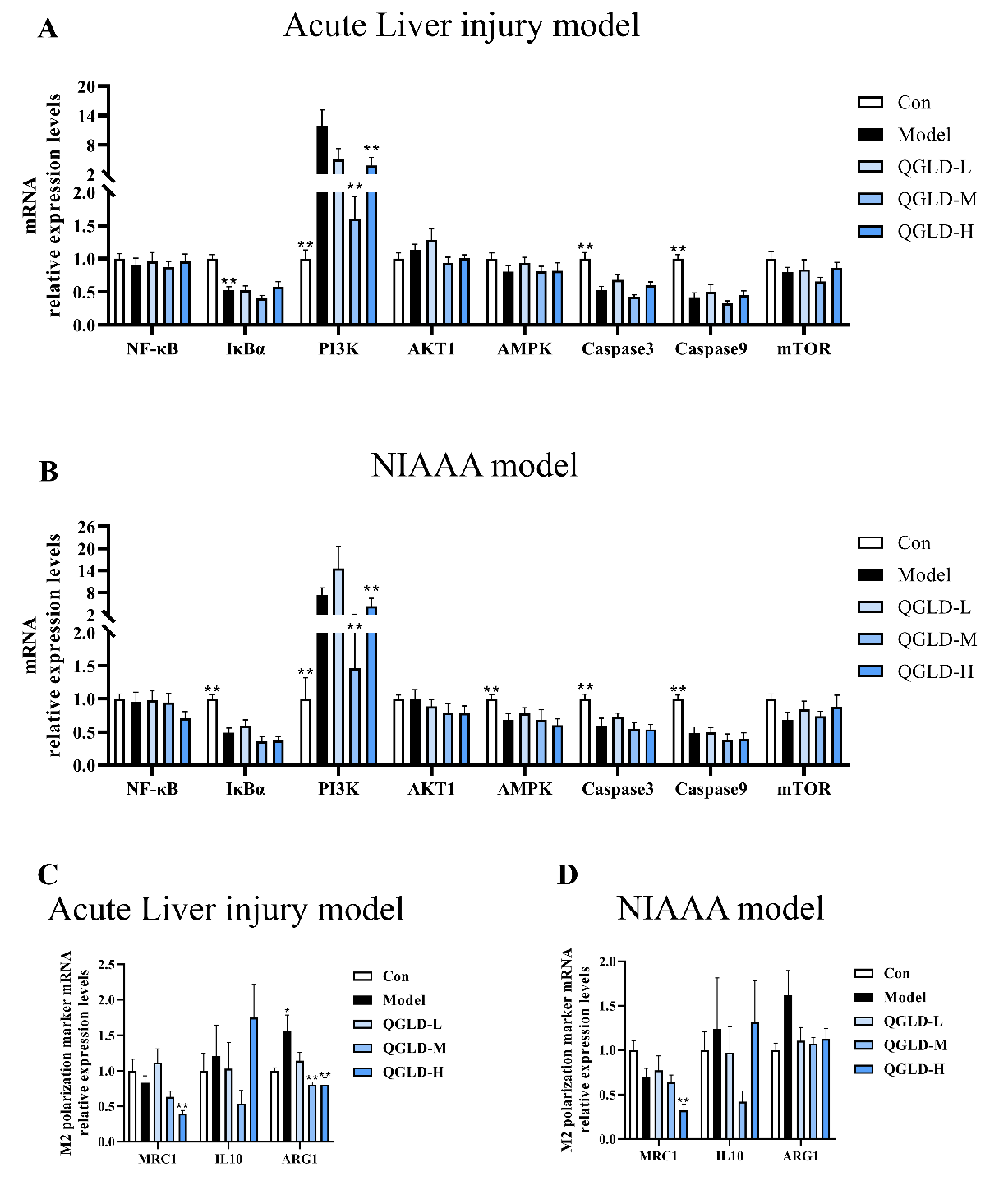


(A) Relative mRNA expression levels of NF-κB pathway, PI3K-AKT pathway, AMPK pathway in the acute liver injury model (n=8). (B) Relative mRNA expression levels of NF-κB pathway, PI3K-AKT pathway, AMPK pathway in the NIAAA model (n=8). (C) Relative mRNA expression levels of M2 polarization markers in the acute liver injury model (n=8). D) Relative mRNA expression levels of M2 polarization markers in the NIAAA model (n=8).**p*<0.05, ** *p*<0.01 vs. the Model group.
